# Isolated Major Aortopulmonary Collateral as the Sole Pulmonary Blood Supply to an Entire Lung Segment

**DOI:** 10.1155/2017/5218321

**Published:** 2017-07-12

**Authors:** Hannah S. Kim, R. Mark Grady, Shabana Shahanavaz

**Affiliations:** Division of Pediatric Cardiology, Washington University School of Medicine in St. Louis, St. Louis, MO, USA

## Abstract

Congenital systemic-to-pulmonary collateral arteries or major aortopulmonary collaterals are associated with cyanotic congenital heart disease with decreased pulmonary blood flow. Though it is usually associated with congenital heart diseases, there is an increased incidence of isolated acquired aortopulmonary collaterals in premature infants with chronic lung disease. Interestingly, isolated congenital aortopulmonary collaterals can occur without any lung disease, which may cause congestive heart failure and require closure. We present a neonate with an echocardiogram that showed only left-sided heart dilation. Further workup with a CT angiogram demonstrated an anomalous systemic artery from the descending thoracic aorta supplying the left lower lobe. He eventually developed heart failure symptoms and was taken to the catheterization laboratory for closure of the collateral. However, with the collateral being the only source of blood flow to the entire left lower lobe, he required surgical unifocalization. Isolated aortopulmonary collaterals without any other congenital heart disease or lung disease are rare. Our patient is the first reported case to have an isolated aortopulmonary collateral being the sole pulmonary blood supply to an entire lung segment. Due to its rarity, there is still much to learn about the origin and development of these collaterals that possibly developed prenatally.

## 1. Introduction

Congenital systemic-to-pulmonary collateral arteries or major aortopulmonary collaterals (MAPCAs) are well recognized and associated with cyanotic congenital heart disease (e.g., tetralogy of Fallot with pulmonary atresia), where there is a decrease in antegrade pulmonary blood flow. In this lesion, aortopulmonary collaterals arise from the systemic arteries and supply majority of the lung parenchyma [1–4]. Acquired aortopulmonary collaterals (APCs) can occur in isolation, secondary to chronic alveolar hypoxia due to inflammatory, neoplastic, or traumatic lung disease. Reportedly, there is also an increased incidence of isolated aortopulmonary collaterals in very low birth weight infants with chronic lung disease. Most of these collaterals are small and inconsequential that regress spontaneously over time [5–10]. In addition, there have been few case reports describing isolated congenital aortopulmonary collateral in those without any lung disease, who presented with congestive heart failure requiring closure via catheterization intervention [11, 12]. We describe here an interesting case of an isolated large aortopulmonary collateral supplying the entire lower lobe of the left lung without dual blood supply, in the presence of a normal intracardiac anatomy or lung disease.

## 2. Case Presentation

An ex 35 weeker, dichorionic-diamniotic twin A, was born to a 26-year-old G1P2 via caesarean section for pregnancy induced hypertension. His birthweight was 2.06 kg and Apgars were 9,9 at one and five minutes of life. At 3 days of life, patient had a murmur and echocardiogram performed showed a mildly dilated left atrium (LA) without any intracardiac lesions. Two weeks later, a follow-up echo showed progression of the LA dilation, left ventricle dilation, and significant pulmonary venous return from the left lower pulmonary vein, without evidence of a PDA. A Computer Tomography Angiogram was performed to look for vascular anomalies. The imaging showed a large anomalous systemic artery arising from the descending thoracic aorta supplying the left lower lobe. Because he was stable on room air and gaining weight appropriately, we deemed it reasonable to intervene in the APC when he was older, to decrease the vascular injury risks of a cardiac catheterization procedure. However, when he was seen as an outpatient, it was reported that the patient was becoming more tachypneic and not gaining weight as well. Thus, he was taken to the cardiac catheterization laboratory for possible closure of the APC.

**Figure 1:**
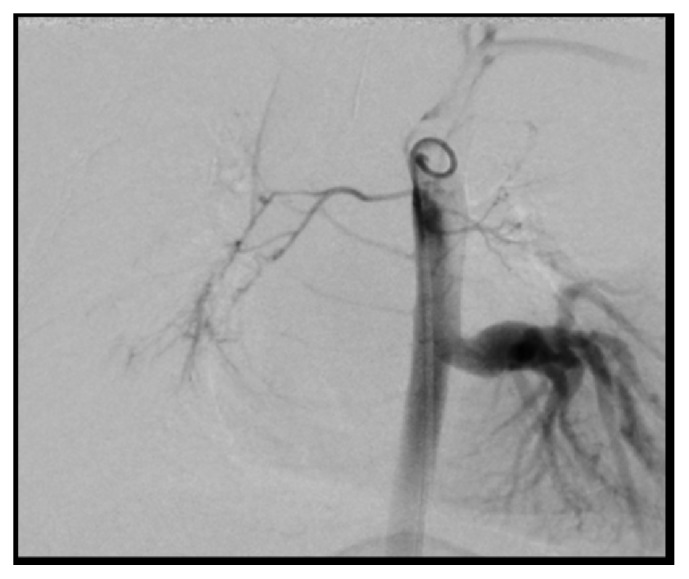
Aortic cineangiogram. Contrast into the descending aorta shows a large aortopulmonary collateral arising from the descending aorta supplying the entire left lower lobe of the lung.

**Figure 2:**
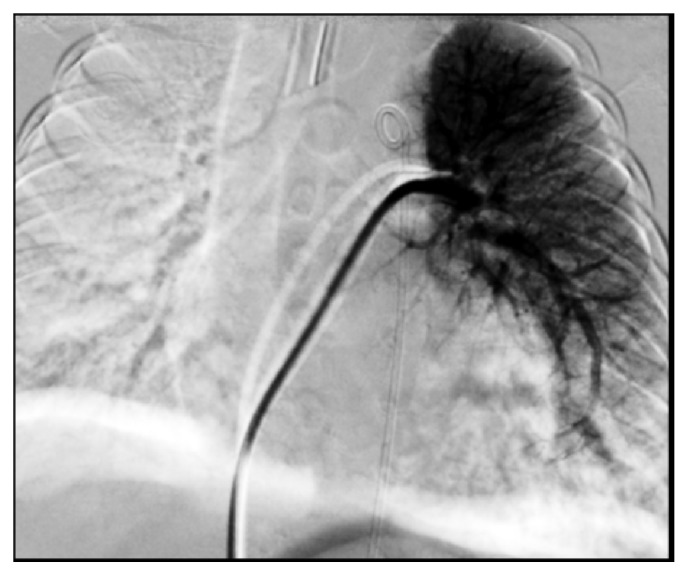
Digital subtraction cineangiography. Contrast injected into the left pulmonary artery shows a small left pulmonary artery perfusing only the left upper lobe of the lung. No flow is seen in the left lower lobe. There is no left lower pulmonary artery.

Data from cardiac catheterization showed normal baseline cardiac index (5.79 L/min/m^2^) with normal intracardiac pressures and no intracardiac shunting. The pulmonary artery pressure (15/10 mmHg, mean 13 mmHg) and transpulmonary gradient (5 mmHg) were normal. Aortic cineangiogram showed a large aortopulmonary collateral supplying the entire left lower lung (Figure 1). A digital subtraction cineangiography performed in the left pulmonary artery (LPA) showed flow only to the left upper lobe (Figure 2). Since the APC was the only source of left lower lung perfusion, it was not closed at this time.

He then underwent surgical unifocalization of the left major aortopulmonary collateral artery onto the left pulmonary artery. The APC itself would not reach the left pulmonary artery and required a segment of the aorta for unifocalization. With that, the aorta was reconstructed with an end to end anastomosis of the descending aorta. Routine postoperative care was taken and he was discharged home safely after 5 days.

## 3. Discussion

The morphologic development of the pulmonary circulation is complex. The intrapulmonary pulmonary arteries arise from the lung buds and the extrapulmonary arteries arise from the proximal portion of the sixth aortic arch. The main pulmonary artery is derived from the truncoaortic sac. At about day 27, the arterial branches of the paired sixth aortic arches form an anastomosis with the intrapulmonary pulmonary arteries. With further development, the branches from the aortic arches enlarge and the descending aorta becomes smaller. When the normal connections fail to develop, collaterals persist.

Major aortopulmonary collaterals may be associated with single ventricle physiology congenital heart diseases such as tetralogy of Fallot with pulmonary atresia. These MAPCAs form early in embryological development due to lack of adequate antegrade pulmonary blood flow in utero. These connections become an additive or sole pulmonary blood supply and thus essential for survival. Though MAPCAs are usually associated with congenital heart diseases, aortopulmonary collaterals that are isolated and occur without intracardiac pulmonary obstruction have also been described. These isolated APCs may develop postnatally in very low birth weight infants who required prolonged ventilator support and in infants with hypoxia due to parenchymal lung disease. The APCs are usually small and regress on their own after treatment of the lung disease. Isolated APCs without cardiopulmonary malformation can also occur and are truly rare. There have only been few case reports in literature with successful transcathether closure after confirmation of another source of pulmonary blood flow to that segment of the lung. To the best of our knowledge, this is the first case report in literature describing an isolated aortopulmonary collateral, with no other cardiopulmonary disease, being the sole blood supply to an entire lobe of the lung. In addition, the patient, albeit premature, had birth weight greater than 1500 g not requiring any ventilator support. Altogether, this suggests an isolated aortopulmonary collateral artery that developed possibly due to faulty embryologic development of the left sided 6th aortic arch or pulmonary arch responsible for development of the LPA, providing supply only to the left upper lobe.

## 4. Conclusion

Isolated aortopulmonary collaterals in a healthy neonate without any other congenital heart disease or lung disease are rare. Our patient is the first reported case to have an isolated large aortopulmonary collateral being the sole pulmonary blood supply to an entire lung segment. Therefore, we did not proceed with coiling of the APC. Due to its rarity, there is still much to learn about the origin and development of these collaterals that possibly developed prenatally.
